# SEIR Modeling of the Italian Epidemic of SARS-CoV-2 Using Computational Swarm Intelligence

**DOI:** 10.3390/ijerph17103535

**Published:** 2020-05-18

**Authors:** Alberto Godio, Francesca Pace, Andrea Vergnano

**Affiliations:** Department of Environment, Land and Infrastructure Engineering (DIATI), Politecnico di Torino, Corso Duca degli Abruzzi 24, 10129 Torino, Italy; francesca.pace@polito.it (F.P.); andrea.vergnano@polito.it (A.V.)

**Keywords:** SARS-CoV-2, COVID-19, SEIR modeling, Italy, stochastic modeling, swarm intelligence

## Abstract

We applied a generalized SEIR epidemiological model to the recent SARS-CoV-2 outbreak in the world, with a focus on Italy and its Lombardy, Piedmont, and Veneto regions. We focused on the application of a stochastic approach in fitting the model parameters using a Particle Swarm Optimization (PSO) solver, to improve the reliability of predictions in the medium term (30 days). We analyzed the official data and the predicted evolution of the epidemic in the Italian regions, and we compared the results with the data and predictions of Spain and South Korea. We linked the model equations to the changes in people’s mobility, with reference to Google’s COVID-19 Community Mobility Reports. We discussed the effectiveness of policies taken by different regions and countries and how they have an impact on past and future infection scenarios.

## 1. Introduction

We present an updated version of the predictive model of epidemic phenomena based on the approach called SEIR (Susceptible-Exposed-Infective-Recovered), widely used to analyze infection data during the different stages of an epidemic outbreak. The SEIR model represents one of the most adopted mathematical models to characterize the epidemic dynamics and to predict possible contagion scenarios. The SEIR model can be useful to assess the effectiveness of various measures, such as lock-down, since the infectious disease outbreak. It is based on a series of dynamic ordinary differential equations that consider the amount of the population subject to contagion, the trend over time of individuals who recover after infection, and the individuals who unfortunately die [1].

This work was carried out during the crucial development phase of the epidemic in Italy (mid-April 2020), with the operational difficulties linked to the impossibility of verifying and validating the databases, and with the difficulty of comparing and calibrating the results with other studies. The purpose, however, is to provide an easy-to-read and useful tool that can help the policymakers, responsible for strategic choices, in assessing the social and economic scenarios related to the development of the epidemic. We are conscious that it is a predictive model which, although based on a scientific approach, is conditioned by a series of intrinsic and endogenous factors that can affect its medium-term reliability. Nevertheless, we are also aware that any political decision potentially lacking any rational and critical evaluations of all the available data risks being based on mere sensations, often dictated by sentimental suggestions [2].

The generalized SEIR model is based on a system of differential equations, as discussed by Peng et al. (2020) [3] in the analysis of the SARS-CoV-2 outbreak in China. The model, that adds complexity to the classical SIR or SEIR models, represents the various conditions of susceptible and infected individuals during an epidemic outbreak (especially quarantined people, who are not able to infect other people during their quarantine). The coefficients of the equations represent the ratios of variation over time of the different categories of individuals, that is, infected, dead, and recovered [4]. These coefficients have often been considered constant [5]. However, they are unable to take into account external influences, such as the actions containing the spread of the infection that may occur at different times during the development of the infection itself or the possible change in health conditions of infected individuals due to pharmacological development. The approach herein discussed introduces time-dependent model parameters. In particular, we assume the infection rate time-dependent, considering that the number of contacts between people, during the lockdown, decreases proportionally to the decrease of their overall mobility, calculated using a big data repository available from Google [6].

The SEIR model is not a novelty in the modeling and forecasting of epidemic phenomena. The main innovation we have introduced concerns the stochastic approach to solve the model and to assess the propagation of the uncertainties of the model solution. This approach is based on a metaheuristic method, the Particle Swarm Optimization (PSO) algorithm, belonging to the family of computational swarm intelligence [7]. In fact, citing the overview made by Parham (2012) [1], the analysis of temporally-forced non-linear epidemic models within stochastic frameworks has received little attention to date due to the complexity of the problem, despite representing the most realistic framework for capturing the behavior of many intrinsically or extrinsically forced infectious diseases. With respect to the standard deterministic approach in solving the SEIR model, the advantage of the PSO approach is that the adaptive exploration of the space domain of the solutions decreases the risk of being trapped into a local minimum and iteratively searches for the global minimum as the final solution. Moreover, the PSO method provides a set of model solutions as probable scenarios calculated by means of a-posteriori probability density distribution.

The SEIR model is applied to the Italian situation at a national level, and at a regional scale, focusing on the most impacted regions in Northern Italy. Like all models, the quality of the observed data and their validation is a crucial node in assessing the reliability of the model results. The data are derived from the official repositories and are composed of infective and recovered individuals, and death cases. We test the approach on other situations such as Spain, which has shown many similarities with the virus diffusion in Italy, and South Korea, which instead represents a completely different scenario. We also explore the main drawbacks of the suggested method, mostly related to the uncertainty of the input data and the complexity in the inclusion into the model of all the external factors (population density and ages, previous diseases, efficiency of the public health system, etc.) which have impacts on the virus diffusion, the recovery rate, and the number of deceases.

The main objective of the work is to improve the classical SEIR model by means of a stochastic solver which identifies a set of possible solutions (or most probable scenarios) predicting the epidemic evolution with the associated uncertainty assessment. Moreover, the mathematical model was modified to adopt a time-dependent infection rate in order to appropriately describe a realistic situation where people’s contacts are not constant in time due to the imposed rules of social distance. The final broad objective is to provide a reliable approach that predicts the epidemic evolution so that the policy makers could undertake both proper initiatives to reduce the contagion and selective actions considering the peculiarity of each region.

## 2. Materials and Methods

### 2.1. Database

The analysis is based on the data collected and made available via a dashboard by the John Hopkins University in the USA. They represent an official database as they collect the data from different official organizations such as the World Health Organization (WHO), the European Centre for Disease Prevention and Control (ECDC), the USA Centers for Disease Control and Prevention, and other organizations. The Italian data are collected entirely through the bulletin of the *Protezione Civile Italiana*. The Italian National Institute of Statistics (ISTAT) was the source of the number of national and regional populations. The number of people living in the studied regions is reported in Table 1.

### 2.2. Overview of the Generalized SEIR Model

The SEIR model simulates the time-histories of an epidemic phenomenon. In its classical form, it models the mutual and dynamic interaction of people between four different conditions, the susceptible (*S*), exposed (*E*), infective (*I*), and recovered (*R*).

The classical SEIR model can be described by a series of ordinary differential equations:

(1) $$\frac{dS\left(t\right)}{dt}=-\beta I\left(t\right)\cdot \frac{S\left(t\right)}{N}$$

(2) $$\frac{dE\left(t\right)}{dt}=\beta I\left(t\right)\cdot \frac{S\left(t\right)}{N}-\gamma E\left(t\right)$$

(3) $$\frac{dI\left(t\right)}{dt}=\gamma E\left(t\right)-\left(\lambda +\kappa \right)I\left(t\right)$$

(4) $$\frac{dR\left(t\right)}{dt}=\left(\lambda +\kappa \right)I\left(t\right)$$

The susceptible (*S*) is the part of the population that could be potentially subjected to the infection: at the initial time, without further information, it is represented by the whole population. The exposed (*E*) is the fraction of the population that has been infected but does not show symptoms yet: it can be called a latent phase, and at this stage, a disease can be infectious, partially infectious or not infectious [8]. The infective (*I*) represents the infective population after the latent period. The recovered (*R*) are the people after healing, and they are generally not reintroduced into the susceptible category if it is supposed that they became immune to the disease. This aspect is strongly debated, as in some countries a second infection of recovered people has been recorded. At this stage of our study, we do not have enough data to include this effect on the model; this would require the introduction of another term in the previous system of equations, including another coefficient that takes into account the re-population of the susceptible compartment. In the classical SEIR model, the *R* category also comprehends the individuals who died of the disease. A characteristic of this model is that the sum of the four categories is equal to the total population (*N*) at any time:

(5) S(t)+E(t)+I(t)+R(t)=N

As can be seen, it does not consider the natural births and deaths of the population during the time span of the disease.

The equations of the classical SEIR model are governed by the parameters *β*, *γ*, *λ* and *κ*. We adopt the symbols used in Peng et al. (2020) [3]. As usual in this field, the following parameters have day^−1^ as a unit of measurement.

- *β* is called infection rate. It is the number of people that an infective person infects each day. It is equal to p, where *b*, or the contact rate, is the number of people an average person enters into contact with each day, and *p* is the probability that a contact provokes the transmission of the disease. In the SEIR model, *β* is the vector which transports people from the *S* category to the *E* category. It is multiplied by the ratio *S*/*N* to avoid counting contacts between two people who cannot infect each other (e.g., because one of them has already recovered, or because both are infective).
- *γ* is the inverse of the average latent time and governs the lag between having undergone an infectious contact and showing symptoms: in the equations, it brings people from the E category to the I category.
- *λ* and *κ* are the recovery rate and the death rate, respectively, and they are united together in a single parameter in the classical SEIR model. They give information about how fast the people may recover from the disease (*1*/*λ* is the average recovery time), and how many of them, unfortunately, die.

Given the complexity of the disease, many authors have implemented different variations of the classical SEIR model, regarding both the equations and the parameters, or managing different fitting techniques to make the model representing the reality as close as possible.

We adopted a generalized SEIR model following the recent publication by Peng et al. (2020) [3], who studied the COVID-19 infection in several Chinese provinces. We applied the model of Peng et al. (2020) to the Italian situation, following this system of equations:

(6) $$\frac{dS\left(t\right)}{dt}=-\beta \left(t\right)I\left(t\right)\cdot \frac{S\left(t\right)}{N}-\alpha S\left(t\right)$$

(7) $$\frac{dP\left(t\right)}{dt}=\alpha S\left(t\right)$$

(8) $$\frac{dE\left(t\right)}{dt}=\beta \left(t\right)I\left(t\right)\cdot \frac{S\left(t\right)}{N}-\gamma E\left(t\right)$$

(9) $$\frac{dI\left(t\right)}{dt}=\gamma E\left(t\right)-\delta I\left(t\right)$$

(10) $$\frac{dQ\left(t\right)}{dt}=\delta I\left(t\right)-\lambda (t)Q\left(t\right)-\kappa (t)Q\left(t\right)$$

(11) $$\frac{dR\left(t\right)}{dt}=\lambda (t)Q\left(t\right)$$

(12) $$\frac{dD\left(t\right)}{dt}=\kappa (t)Q\left(t\right)$$

This SEIR model adds some features to those of the classical SEIR model (Equations (1)–(4)):

It supposes that the susceptible population decreases thanks to lockdown policies and improvements in public health behaviors, such as wearing face masks. Each day, several individuals (S·α) passes from the susceptible category to the protected category (*P*), being *α* the protection rate.

It adds the category of quarantined people (*Q*). The passage from the infective to the quarantined category is done through the parameter *δ*, which is the inverse of the average time required to quarantine a person with symptoms: this happens usually after the person has been tested positive. The quarantined people are hence excluded from the infective category (*I*) because they are supposed not to have had any contact with others. The quarantined category matches the “active confirmed cases” in Italian official datasheets, and, according to the common habit of quarantining positive people, it is true also for data from most developed countries. This is a critical point for the system of equations, that according to us should be better defined. In fact, an infected individual is not automatically quarantined, because the authorities were often unable to test enough people while keeping pace with the spread. This is especially difficult because many people do not develop symptoms at all, but can transmit the infection to others. So, we think that *δ* also contains some information about the percentage of the detected infective people. A study from Calafiore et al. (2020) [5] proposed the introduction of an additional parameter to better understand this issue.

It separates the categories of recovered (*R*) and dead (*D*) people, linked to the quarantined category through the *λ* and *κ* parameters, the cure rate, and the mortality rate respectively. *λ* and *κ* are time-dependent because the health system can improve its capability to treat people over time, e.g., with the introduction of a new therapy. Based on the data collected from Chinese reports in Peng et al. (2020) [3], which suggested an exponential evolution of the two parameters, we constrained *λ* and *κ* to fit an exponential trend. Similarly to Cheynet (2020) [9], the assumption is that the death rate should become closer to zero as time increases, while the recovery rate converges toward a constant value:

(13) $$\lambda (t)=\lambda_{0}\left[1-exp\left(-\lambda_{1}t\right)\right]$$

(14) $$\kappa (t)=\kappa_{0}exp\left(-\kappa_{1}t\right)$$

The *λ*_0_ represents the final asymptotic value of the cure rate. It is related to the health system’s ability to tackle the infection after adapting to the new outbreak and depends also on other factors like the good health of the citizens. *λ*_1_ is related to how fast the adaptation to the emergency was. At the beginning of April, South Korea was already in a post-peak phase of the disease spread. From our initial tests, Equation (13) did not ideally match the data of South Korea, probably because of the more complicated trend, compared to other countries. Therefore, only for the South Korean model, the λ parameter was not constrained by an exponential law. We increased the degrees of freedom of its trend by imposing a sinusoidal law:

(15) λ(t)=a·sin(bt+c)+d·sin(et+f)

The fitting of the six parameters was performed by rearranging Equation (11):

(16) λ(t)=dR/(dt·Q)

where *R* and *Q* are the data series of recovered and quarantined people. This approach was successful in providing a better fitting of the model prediction to the data.

The parameter *κ*_0_ represents the initial value of the mortality rate. The *κ*_0_ value is related to the initial health system’s ability to tackle the infection and depends on the good health of the citizens. The *κ*_1_ value measures how the rate has changed with time. The mortality rate is supposed to decrease over time, and the higher *κ*_1_ is, the faster this decrease.

We introduced an improvement to the *β* parameter, compared to Peng’s model, that is, its time dependency. Since the infection rate *β* is proportional to the contact rate *b*, as stated before, we estimated the variation in the contact rate according to the recent publication of Google’s COVID-19 Community Mobility Reports [6], a database built on GPS data collected from mobile devices with the “Location History” option turned on. It provides data about the reduction in the mobility of people over the recent few months. For each investigated region, we calculated the average mobility decrease over time, and we fitted the curve with a second-order polynomial trend line. Then, we constrained *β* to be proportional to that specific trend line. In a preliminary test on a simple data set, we noticed how the introduction of this constraint allowed the model to obtain a better data fitting. Therefore, we applied this approach to the SEIR model to ensure good data fitting for all the regions and countries investigated. We noticed that the mobility data of South Korea did not show a significant decrease in people’s mobility, because the Government adopted a different strong approach in lockdown policies compared to most European countries. Strict lockdowns were not imposed, but efforts were addressed to track the infection spread at the early stages, with tight controls and strict quarantine protocols for infected individuals. For the 30 days of model prediction, the time-dependent *β* parameter had the same value of the last observed day (i.e., mid-April) since we did not have reliable predictions about future mobility. This means that the lockdown policies will continue, or the reopening of business will be made paying close attention to health protection procedures.

**Figure 1:**
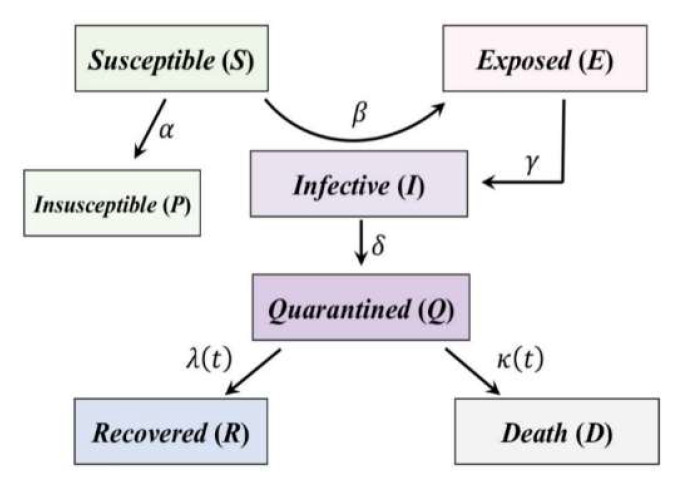
Generalized SEIR (Susceptible-Exposed-Infective-Recovered) model scheme (modified from [5]).

The generalized SEIR-model scheme is described in Figure 1. The expected evolution of the equation terms is: the susceptible category decreases over time, feeding the exposed (*E*) category through the beta parameter *β* and the protected (*P*) category through the α parameter; the latter represents the part of the population who for various reasons becomes insusceptible to the disease. The exposed (*E*) category is only a temporary category: its individuals pass into the infective (*I*) category after a latent time (1/*γ*), on average. The infective category generates newly infected people over time, removing them from the susceptible category. The detected infected individuals are quarantined (*Q*) to avoid spread. Then, they evolve into recovered (*R*) or death (*D*) cases, according to various causes, like health care system effectiveness, age, co-morbidity of other diseases. It is important to note that the most reliable data series provided by national agencies are *Q*(*t*), *R*(*t*), and *D*(*t*). The fitting of these data in the structure of the generalized SEIR model allows the trend of other categories to be estimated with some degrees of uncertainty, as well as their prediction for the subsequent 30 days.

The main outputs of the model are the following data series:

- *S*, target time-histories of the susceptible cases,
- *E*, the target time-histories of the exposed cases,
- *I* the target time-histories of the infective cases,
- *Q*, the target time-histories of the quarantined cases,
- *R*, the target time-histories of the recovered cases,
- *D*, the target time-histories of the death cases,
- *P*, the target time-histories of the insusceptible cases.

The *α*, *β*, *γ*, *δ*, *λ* and *κ* parameters can be considered a major output of the model. In particular, the evolution over time of *λ* and *κ* could provide information about the changes in the health system response to the contagion. *β* is also time-dependent, and it is constrained to be proportional to the people’s mobility trend extrapolated from Google’s big data. The *α*-value is also related to policies, although it is not closely related to a precise aspect of the government strategies, as the *β* parameter. The *α*-value was not forced to follow any particular law during the modeling since we tested no significant improvement by modifying it from a constant to a time-dependent parameter.

The various researches about SEIR-like models applied to the current SARS-CoV-2 epidemic have introduced minor or major changes to the classical SEIR model. It can be useful to overview the values of the main parameters, to define a realistic range of values, and to understand their meaning. We used the coefficient values found in literature to set the lower and upper boundaries of the parameters in our modeling. The values of these parameters are related to different methods. Therefore, the bibliographic research summarized in Table 2 should be only considered as a qualitative benchmark. We highlight some peculiarities of the various studies.

The study by Calafiore et al. [5] presents the values of the parameters also for each Italian region. The peculiar SEIR model used here introduces two new parameters: *α* (with a meaning different from ours) and *ω*. *α* represents how many times the real number of infected people is higher than the number of detected infected people, and it is estimated to be about 63. The *ω* parameter defines a fixed percentage of total people susceptible to the disease, and according to this model, it is about 0.124 (12%).

One of the WHO reports [10] shows a list of estimates of Serial Interval, which is the average time between infection and subsequent transmission. We reported the latter since it is closely related to the inverse of *β*. It ranges from 5 to 9 days, according to the various studies considered.

Dandekar et al. [11] calculates the change over time of a term, called quarantine strength (*Q*(*t*)), fitted thanks to a neural network-based approach.

Shaikh et al. [12] considers as separated the asymptomatic and symptomatic categories.

The most interesting feature of the model proposed by Lin et al. [13] is the time-dependent *β*, implemented with a different design compared to our model:

(17) $$\beta (t)=\beta_{0}\left(1-\alpha \right){\left(1-\frac{D}{N}\right)}^{k}$$

where *α* is a stepwise function that represents the governmental action, estimated to range from 0 to 0.85 according to the strength of lockdown policies. while *k* is the citizen response, estimated to be about 1100.

Iwata et al. [14] proposes a model which does not fit real data but investigates possible scenarios deriving from a different combination of parameters. Their range is reported as a credible range reference.

### 2.3. Implementation of the SEIR Model with a Stochastic Approach

The model equations were implemented on the basis of the MATLAB code provided by [9], available in Matlab File Exchange. The data are extracted from the official repository and are composed of: confirmed, recovered, and death cases (*Q*, *R*, and *D*, respectively). These values represent the initial assumptions, while the parameters *α, β, γ, δ, λ,* and *κ* are the problem unknowns. The differential equations are numerically solved by means of the Runge–Kutta method.

The standard approach of the source code uses as default a least-square fitting solver to match the observed data and the calculated response (of *Q*, *R*, and *D*). At the beginning of modeling, the initial values of the six parameters are given as first esteem. Then, their values are calculated following a least-square solver that considers the observed data (*Q*, *R*, *D*) with time.

We modified the standard release of the code by introducing a new solver, the PSO algorithm, belonging to the family of computational swarm intelligence (population-based nature-inspired metaheuristics) [15,16]. This optimization solver minimizes an objective function, which is set to decrease the misfit between observed data and calculated responses of *Q*, *R*, and *D* by varying the six parameters, i.e., the problem unknowns. The main advantages of the stochastic approach over the deterministic method to solve the SEIR model are briefly discussed. The adaptive exploration and exploitation of the search space of the model solutions avoids the risk of being trapped in some local-minima solutions and also enhances the independence from the initial assumption of the six parameters which could bias the final solution. The solution search-space is sampled by a set of 200 particles, representing the possible solutions, which are randomly initialized. The adaptive behavior and the convergence and stability of the final solution are ensured by using a PSO variant, the hierarchical PSO with time-varying acceleration coefficients (HPSO-TVAC) [17]. Convergence was achieved in 150 iterations. Each run of 150 iterations was repeated for 50 trials to test the variability of the solutions due to the random initialization of the parameters. Finally, the trial showing the minimum normalized root mean square error (NRMSE) was selected as the best solution. The solutions from the remaining trials were a-posteriori evaluated with their probability density distribution. The solutions within 5% of the minimum NRMSE were chosen as representative of other probable scenarios.

Deploying a stochastic approach increased the computational cost of the modeling. Therefore, the code was parallelized to be run on multiple cores. The simulations ran on the academic High Performance Computing (HPC) cluster of Politecnico di Torino. The sustained performance of the cluster is globally 20.13 TFLOPS and the CPU model of one node is 2x Intel Xeon E5-2680 v3 2.50 GHz 12 cores. We adopted 24 cores of a single node.

## 3. Results

Here we present the time series obtained by the standard deterministic approach and the data series obtained by the stochastic approach, based on the Particle Swarm Optimization (PSO) algorithm. First, we analyze the Italian framework at a national and regional scale. Then, we provide the results of SEIR modeling for two other countries: Spain and South Korea. Spain was chosen because the epidemic spread is similar to the Italian one, while South Korea represented a testing data set as the epidemic peak had already been overcome. The final NMRSE of the modeling and the values of the SEIR coefficients are supplied in Table 3.

**Figure 2:**
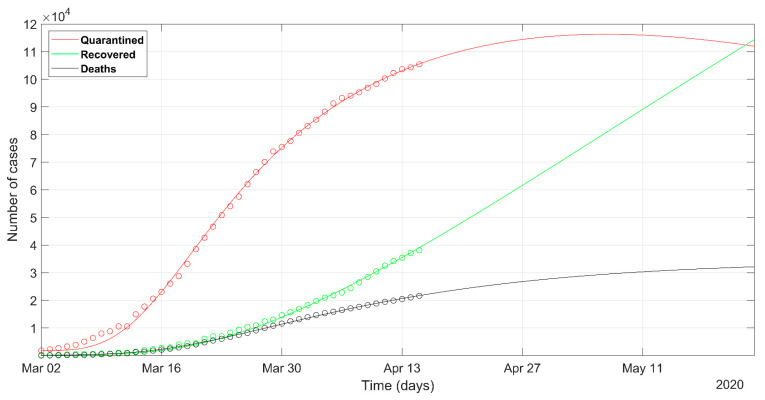
Observed data (**circles**) of quarantined (**red**), recovered (**green**), and deaths (**black**) for Italy, updated on 15 April 2020. The continuous lines refer to the predicted evolution in 30 days according to the SEIR model solved with the deterministic approach.

**Figure 3:**
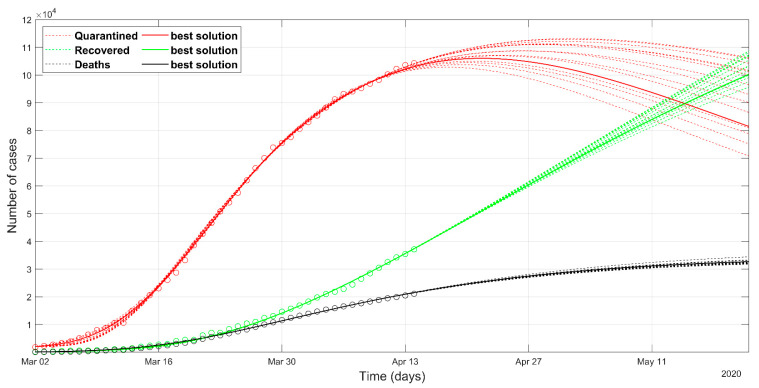
Observed data (**circles**) of quarantined (**red**), recovered (**green**) and deaths (**black**) for Italy, updated on 15 April 2020. The continuous lines refer to the predicted evolution in 30 days according to the SEIR model solved with the stochastic approach. The solid line refers to the best Particle Swarm Optimization (PSO) solution, the dashed lines refer to the most probable solutions (i.e., the solutions within 5% of the minimum normalized root mean square error (NRMSE)).

The prediction of the Italian situation according to the deterministic solver shows the trends given in Figure 2. The result of the PSO approach is shown in Figure 3. Observed data of quarantined, recovered, and death cases are marked in red, green, and black circles, respectively. The individuals tested positive and placed in quarantine (at home, or hospitalized, or in intensive care) are plotted in red color. The sum of quarantined, recovered, and deaths, at a certain date, represents the total confirmed cases at that moment.

The Italian data set starts from 1st March, because we start to model from the day when the confirmed cases were 1% of the maximum counted cases. The predicted curves are plotted with solid lines in Figure 2 and Figure 3. The set of most probable PSO solutions (within 5% of the minimum NMRSE) is plotted with dashed lines in Figure 3. The predicted peak in the red curve represents the status in which the rate of recoveries becomes greater than the rate of infection. It reflects the most relevant impact on the health system, because the numbers of quarantined people, both at home and in the hospitals, are at their maximum. For the Italian situation, the maximum number of the predicted quarantined cases is expected after 27th April according to the deterministic approach and some days before the day according to the best solution of the PSO approach. The curves of recovered and deceased cases in Figure 2 and Figure 3 are similar. The final NRMSE was 0.035 and 0.043 for PSO and deterministic modeling, respectively (Table 3).

**Figure 4:**
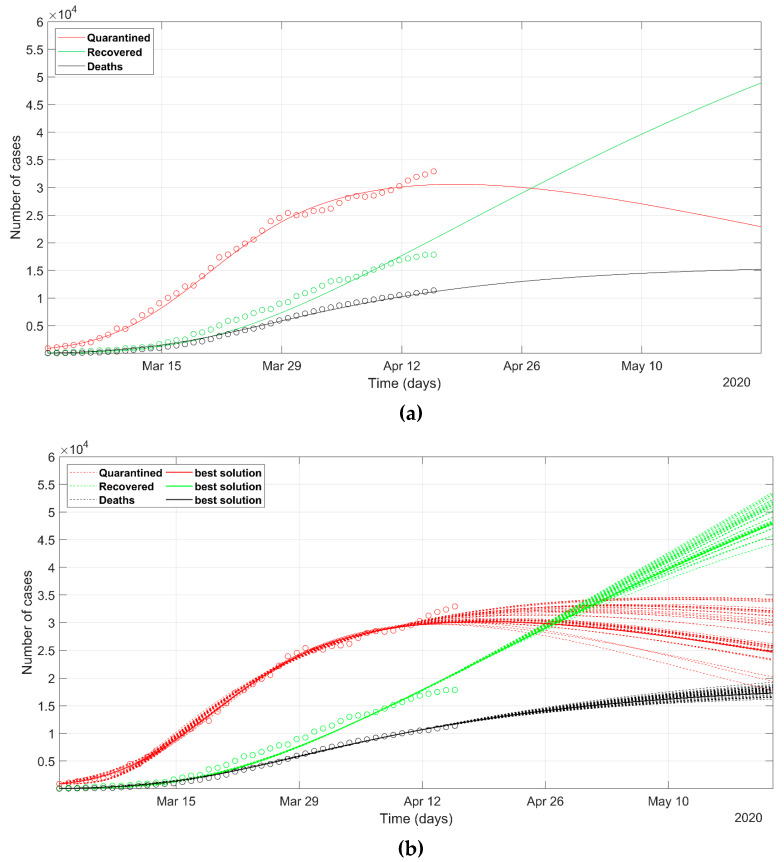
Observed data (**circles**) of quarantined (**red**), recovered (**green**), and deaths (**black**) for the Lombardy region, updated on 15 April 2020. The continuous lines refer to the predicted evolution in 30 days according to the SEIR model solved with (**a**) deterministic approach, (**b**) stochastic approach. In (**b**) the solid line refers to the best PSO solution, the dashed lines refer to the most probable solutions (i.e., the solutions within 5% of the minimum NRMSE).

**Figure 5:**
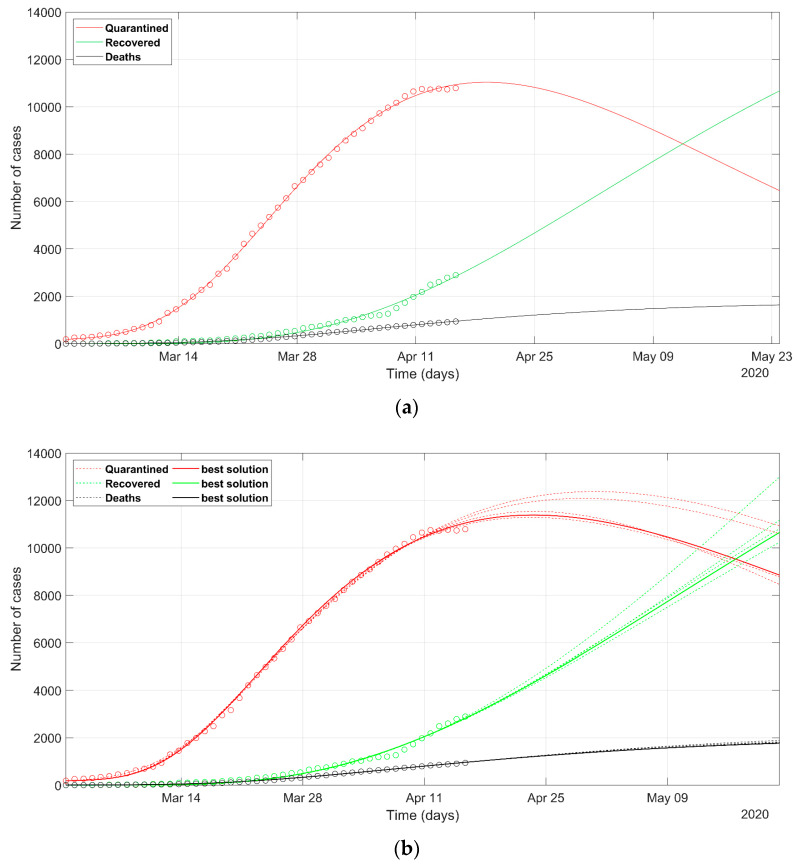
Observed data (**circles**) of quarantined (**red**), recovered (**green**), and deaths (**black**) for Veneto region, updated on 15 April 2020. The continuous lines refer to the predicted evolution in 30 days according to the SEIR model solved with (**a**) deterministic approach, (**b**) stochastic approach. In (**b**) the solid line refers to the best PSO solution, the dashed lines refer to the most probable solutions (i.e., the solutions within 5% of the minimum NRMSE).

**Figure 6:**
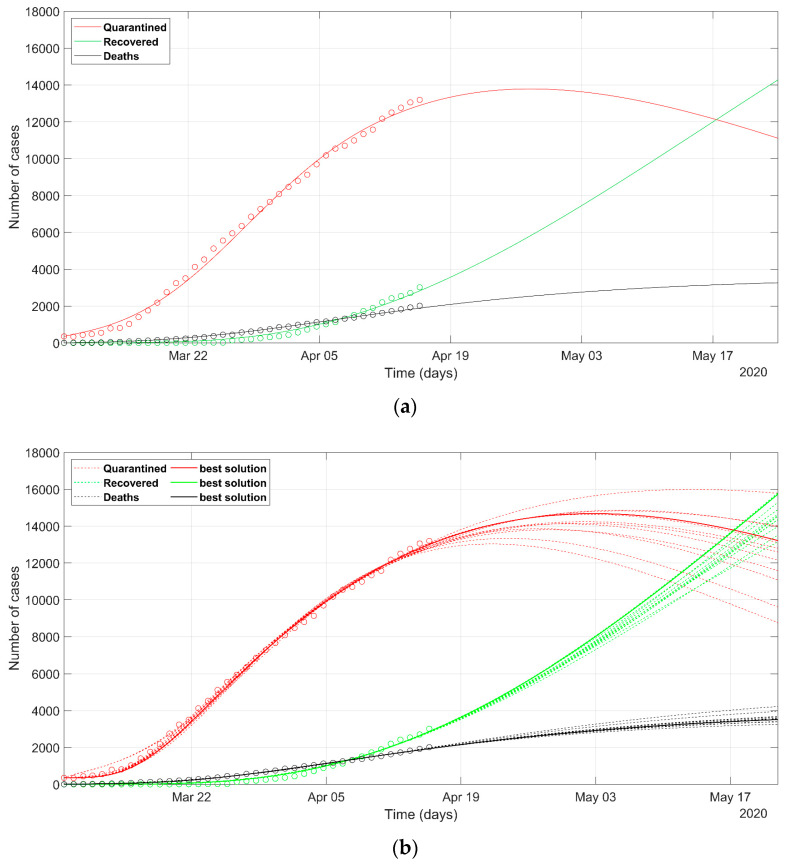
Observed data (**circles**) of quarantined (**red**), recovered (**green**), and deaths (**black**) for the Piedmont region, updated on 15 April 2020. The continuous lines refer to the predicted evolution in 30 days according to the SEIR model solved with (**a**) deterministic approach, (**b**) stochastic approach. In (**b**) the solid line refers to the best PSO solution, the dashed lines refer to the most probable solutions (i.e., the solutions within 5% of the minimum NRMSE).

The analysis of the situation of Lombardy, Veneto, and Piedmont regions is depicted in Figure 4, Figure 5 and Figure 6, respectively. Lombardy was strongly impacted by SARS-CoV-2, as at the end of March, nearly 40,000 novel infected cases and more than 5000 deaths were recorded in a population of 10 million. On the contrary, the Veneto region evidenced 7000 cases and about 300 deaths in a population of 5 million people.

The prediction of the situation in Lombardy, according to the deterministic approach (Figure 4a), appears rather optimistic, as the trend of the quarantined should start to decrease in a few days (red solid line). This probably does not reflect the evolution of the true situation in that region, even if the rate of the recovered generates positive feelings. If we look at the most probable scenarios predicted according to the PSO analysis (dashed lines in Figure 4b), the wide spreading of the trend of the quarantined indicates how any eventual less-restrictive policy must be evaluated with great care in the next days. The set of most probable solutions from PSO presents a wide range of solutions, wider than that for Italy (Figure 3). The final NRMSE was 0.062 and 0.061 for PSO and deterministic modeling, respectively (Table 3).

Figure 5a,b shows the SEIR model prediction for the Veneto region, according to the deterministic and PSO approaches, respectively. While the predicted recovered and death cases are in accordance, the curves of quarantined cases present a slightly different estimate of the predicted peak, which is comprised between 11th and 25th April. The final NRMSE was 0.035 and 0.04 for PSO and deterministic modeling, respectively (Table 3).

The SEIR modeling for the Piedmont region is shown in Figure 6a,b, where the solution using the deterministic and PSO prediction are reported, respectively. The scenarios predicted from PSO are a little worse than those of the deterministic solutions. However, the observed data of Piedmont yield a wide range of probable solutions (dashed lines), which can be overlapped to the deterministic solution in some cases. The final NRMSE was 0.056 and 0.05 for PSO and deterministic modeling, respectively (Table 3).

**Figure 7:**
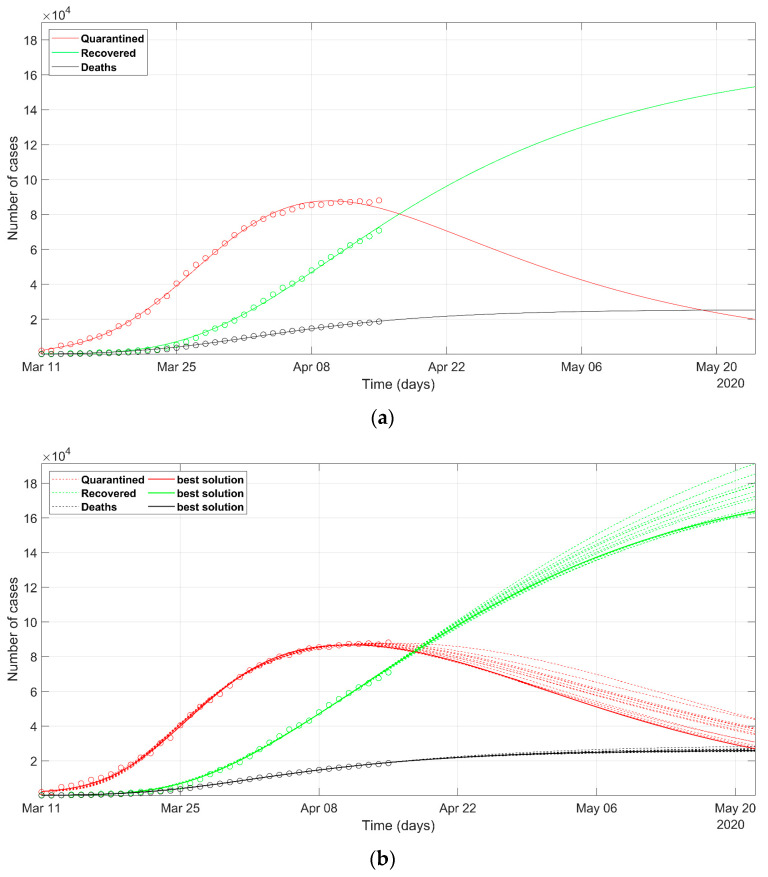
Observed data (**circles**) of quarantined (**red**), recovered (**green**), and deaths (**black**) for Spain, updated on 15 April 2020. The continuous lines refer to the predicted evolution in 30 days according to the SEIR model solved with (**a**) deterministic approach, (**b**) stochastic approach. In (**b**) the solid line refers to the best PSO solution, the dashed lines refer to the most probable solutions (i.e., the solutions within 5% of the minimum NRMSE).

The epidemic situation in Spain is shown in Figure 7. The crisis exploded in a few days after the Italian collapse, as the direct consequence of the delay in undertaking restrictions in business and social activities to limit the spreading of the infection. At this stage of the evolution of the phenomenon in Spain, after one month, the results obtained by the deterministic approach forecast a trend of the recovered that seems very optimistic if compared with the Italian situation. We can assume that the Spanish health system will react promptly to the last round of the infectious. The result of PSO modeling is shown in Figure 7b. The final NRMSE was 0.046 and 0.052 for PSO and deterministic modeling, respectively (Table 3).

**Figure 8:**
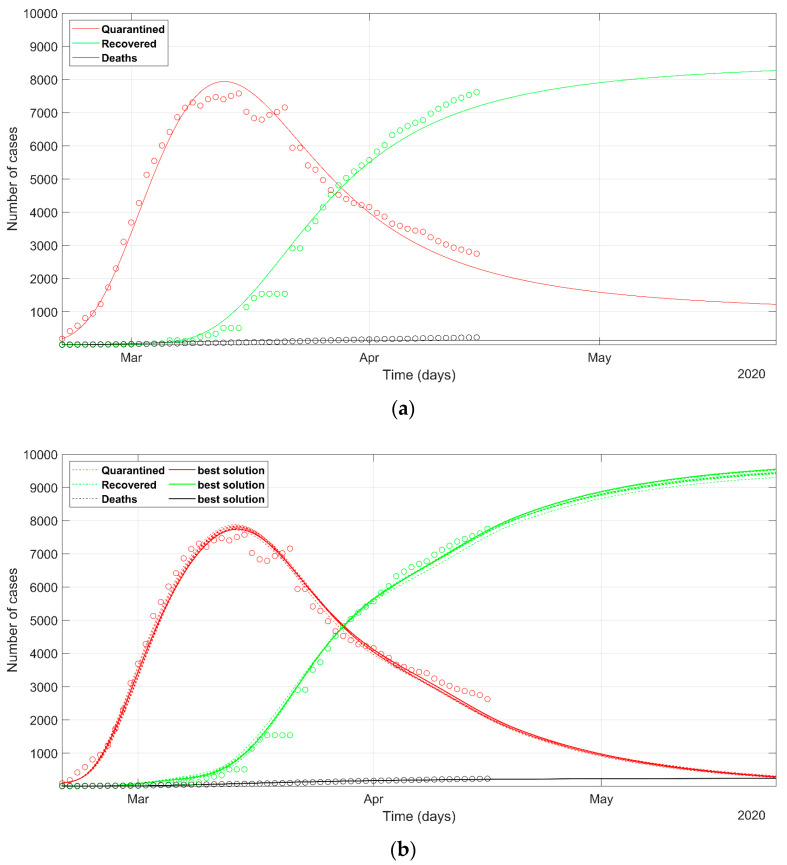
Observed data (**circles**) of quarantined (**red**), recovered (**green**), and deaths (**black**) for South Korea, updated on 15 April 2020. The continuous lines refer to the predicted evolution in 30 days according to the SEIR model solved with (**a**) deterministic approach, (**b**) stochastic approach. In (**b**) the solid line refers to the best PSO solution, the dashed lines refer to the most probable solutions (i.e., the solutions within 5% of the minimum NRMSE).

The trend of the cases and the predicted response of South Korea’s situation is presented in Figure 8. The analysis of the data about South Korea is useful to look at the Italian situation with respect to a country where, for many reasons, the infection was limited, even if the crisis seemed very dramatic at the early stage. The abrupt changes of the recovered and quarantined trend required a careful analysis of the SEIR coefficients and their temporal variation. The final data fitting was indeed not ideal because of the marked oscillations in both the time-series of quarantined and recovered cases. The final NRMSE was 0.074 and 0.078 for PSO and deterministic modeling, respectively (Table 3).

## 4. Discussion

We adopted a generalized SEIR model to offer a quantitative overview of the complex analysis of the SARS-CoV-2 epidemic, meanwhile the disease is still running. The parameters were fitted in a least-square sense with a deterministic approach, and then with a stochastic approach, using a Particle Swarm Optimization (PSO) algorithm, a novelty in the field of epidemiological studies.

The analysis of the results from the stochastic approach gives an overview of the most probable scenarios selected among the solutions within 5% of the normalized root mean square (NRMSE) of the best solution. For each investigated area, we performed 50 trials of PSO simulations and from 5 to 15 trials belonged to the most probable set. It is noticeable that the predicted model responses led to an approximately equivalent the data fitting (normalized with respect to the mean value within an L_2_-norm < 0.05). The probable scenarios sometimes presented a wide range of possible solutions because of the intrinsic setting of the stochastic approach. The different scenarios were achieved thanks to a deeper investigation of the model-space domain where the solutions are not driven and influenced by the initial guess of the SEIR model coefficients. One of the main limits of the deterministic approach, instead, is that the results are biased by the selection of the starting point of model parameters.

The data seem to confirm that while Lombardy and Piedmont applied similar approaches to social distancing and retail closures, Veneto’s strategy applied a much more proactive effort to limit the contagion, by means of extensive testing of symptomatic and asymptomatic cases early on, jointly with an effective tracing of potential positives. The different actions undertaken by the Regions are well depicted in the future trend of the model, with evident advantages in an earlier end of the infection spreading in Veneto (compare Figure 4 and Figure 6 with Figure 5). In fact, the peak of quarantined in Veneto lies before those of Lombardy and Piedmont. The descending curve in Veneto has a sharper trend than that of the other two regions. Moreover, in Veneto, the predicted fatalities are ten times lower and the recovered are five times lower than those in Lombardy.

The behavior of Piedmont (Figure 6) deals with a peculiar trend, introducing a time-delay of the recovered (green curve) with respect to the death cases (black curve), since the number of the recovered in the month of March is always lower than the deaths. This is because the intersection of the trends of recovered and death cases is reached later than the other regions herein analyzed, i.e., 7th April. This probably resulted from the regional testing policy that tested (and counted as confirmed) only patients with severe symptoms or at high risk. The high rate of fatalities that occurred in March was also due to the unexpected stress on the health system and the scarcity of intensive-care units. Differently, Lombardy and Veneto experienced a higher rate of recovered patients at the early stage of the epidemic outbreak.

A recent analysis [18] has pointed out how, according to the guidance from public health authorities in the central government, Lombardy’s actions involved a more conservative approach mainly focusing on the symptomatic cases. They also suppose that the set of policies enacted in Veneto minimized the burden on hospitals and minimized the risk of spreading in medical facilities. Veneto’s strategy tried to prevent the diffusion by capillary actions at the local scale, to limit the contagious with additional measures in the hot spots of the infection at the early stage of the epidemic.

The expected trend of these regions was controlled by many factors outside the control of policymakers, including Lombardy’s greater population density and a higher number of cases at the explosion of the crisis. Nevertheless, the different public health policies at the early stage of the epidemic phenomena also had an impact, and it seems that tailored capillary actions, as in the example of Veneto, obtained better results than applying only a regional lockdown. The difference in the approaches can be underlined by observing that many municipalities or provinces declared “red zones”, where, due to high transmission of the infection, additional restrictive measures were introduced, compared to the rest of the regional territory. In the red zones, the different policies acted in response to local epidemiological situations. Instead, in Piedmont and Lombardy, no red zone was established, but restrictive individual distancing measures were regulated on a regional scale. According to the evidenced results of different policies, in the next phase of governmental policies, the reopening of business and activities should be tailored to the local situations, focusing on the organization and integration of all figures of the health system. In particular, the central government should require from the regions an effort to provide local epidemiological data in real-time, to lockdown only limited areas, while the reopening of regional-scale business can be eased.

The estimated parameters that regulate the equations of the SEIR model are reported in Table 3. For the parameters obtained with stochastic approach, the best-solution is shown in bold, while in brackets the mean and the variance of the solutions within 5% of the minimum NRMSE in brackets. In Table 3 we compare the parameters among different regions, and between the stochastic and deterministic approach. Both approaches provided models that fitted the observed data with good accuracy, although the stochastic approach has, in general, a slightly lower NRMSE.

The parameters calculated with PSO are reported with the best-solution value, mean, and variance. We can observe that *α*, *β*, *γ* and *δ* had a high variance due to the intrinsic variability due to the stochastic approach. Sometimes the best solution is not aligned with the mean value. *λ* and *κ* values, instead, are strictly gathered around the mean in almost all PSO solutions, hence the low variance. This is explained by considering that, since the number of parameters is higher than the available data series (*Q*, *R*, and *D*), the problem is underdetermined, so that the stochastic approach can find more than one series of parameters which fits the data within an acceptable misfit. Then, *λ* and *κ* do not show large variability among the most-probable scenarios because they govern the equations that correlate *Q* with *R* and *D*, that is the official data series. Therefore, the estimated *λ* and *κ* were always found in the same region of the search space of solutions. South Korea and Veneto show the highest recovery rates (*λ*_1_) with values around 0.05, followed by Spain (0.044). This confirms the reports which praise the Veneto model, because its administration had the capabilities of testing more quickly than other Italian regions, and the family doctors worked in a stronger synergy with the health structures. It also evidences a lower death rate (*κ*_0_), probably due to the better health system efficiency to treat patients, but also to the greater number of tests. In both data and policies, the Veneto region is more like South Korea than other parts of Italy. These aspects had an impact on the outbreak of the epidemic, as can be seen comparing Figure 4, Figure 5 and Figure 6: The Veneto region is more likely to reach the peak of active cases (*Q*) before the other regions.

Even though the PSO results may seem to provide a wide range for the SEIR parameters, we stress two important aspects:

- as we already stated, the problem is underdetermined, so it is preferable to have an acceptable range of values than a unique point value, that could result in being uncertain, as could happen considering only a deterministic approach solution;
- the set of possible predicted scenarios, although related to different solutions with different sets of parameters, are quite similar, thus offering an acceptable level of variability of future predictions.

While it would be very useful to estimate a more narrow range of parameters like the infection rate *β* or the latent time (1/*γ*), this is beyond the goals of our study, and the topic is being explored by researchers who focus also on the clinical aspects of the disease.

The model has some limitations, as previously discussed. We summarize them to highlight possible needs in the further development of the modeling.

- We have currently not sufficient information to say that, after recovery, an individual becomes totally immune to the disease, but we made this assumption: the model did not allow the passage from the recovered category to the susceptible category.
- The model does not consider that the exposed category may have a partial infection ability, as described in Shi (2020) [8], nor distinguishes symptomatic from asymptomatic people, as studied in Shaikh (2020) [12].
- The model does not consider the testing differences between different health system structures and country policies.
- While Italian and Spanish data are well fitted, the South Korean data fitting presents some issues. This evidences that different policies between countries can induce different trends in the spread of the epidemic and that the models should be adapted to different situations, with the introduction or removal of parameters. This would be especially valid in analyzing the situation of the least developed countries, that are not able to afford strict lockdown policies like the developed countries.
- Except for the death rate parameter, the model does not have a strong link to the health resiliency of citizens. The death rate parameter could also be related to external factors like air pollution, which makes people more sensitive to respiratory diseases [19].
- The introduction of Google’s COVID-19 Community Mobility Report represents a constraint that was easily implemented in the model. Further studies on the quality of those data and a rigorous implementation could represent a novel and interesting research topic.

We think that many of these issues could still remain open, but the critical point of the study is not to determine exactly in which way each external factor influences the trend of infectious cases, because we are analyzing a multifaceted problem from a global point of view. Moreover, the official data we consider are suspected to be not enough accurate to be the basis of a very detailed study.

## 5. Conclusions

We applied two different approaches for solving the equations of the SEIR model to describe the evolution of the epidemic phenomenon in Italy and in the most impacted regions of the North of Italy (Lombardy, Veneto, and Piedmont). We considered all the possible available data on the 15 April 2020. The main findings indicate that the deterministic approach is not appropriate to explore the possible solutions of the space-domain because the mathematical problem is underdetermined. We recommend fitting the data of this epidemic using a stochastic approach, such as the PSO method. Taking advantage of the PSO approach, we estimated different scenarios for a 30-day epidemic evolution. Every scenario refers to a different set of parameters estimated by the algorithm. The predicted scenarios are fairly similar and suggest that every Italian region will reach the peak of the epidemic by mid-May. The influence of the time-varying infection rate *β* on the model prediction may open interesting discussions about the effect of lockdown policies on the evolution of the epidemic in the near and far future.

Because the model was provided rapidly and the study was performed during the international emergency, we did not explore further the implications of different “reopening” scenarios. We can say that, if the *β* parameter remains at current values, e.g., if the lockdown policies are maintained or, better, the reopening of business is done with particular attention to health safety procedures, the prediction of the trend of the recovered and deaths could be considered reliable, with the approximations and the uncertainties that the PSO model has pointed out. At the Italian level, despite the great dispersion in the prediction of the quarantined and recovered cases, the number of deaths will reach a number of around 33,000–35,000 cases at the end of May and the number of active cases will gradually decrease. This prediction cannot consider the impact of future decisions on social distancing.

The data and the model predictions confirm that some valuable lessons should be learned from the approaches of South Korea, which was able to contain the contagion very soon before a wide spread of the infection. The Veneto region was one of the best examples in Italy about how integrated and synergic regional policies in social distance and the health system can tackle the epidemic, and its epidemiological scenario is now more optimistic than those of Lombardy and Piedmont. We stress that tailored actions provide much better epidemiological outcomes than wide lockdowns, keeping in mind also the example of South Korea.

The main purpose of our work was to provide a fresh discussion and new tools able to support the policymakers in their decision about the action to minimize the impact of the disease. The analysis demonstrates that, because the Italian health care system is highly decentralized, different regions managed different policies, which highly influenced the evolution of the epidemic in its first months: the data and the model prediction well reflected the different approaches taken by Lombardy and Veneto, two regions with similar socio-economic tissue. The overall lesson that could be learned from this analysis goes beyond the mathematical modeling itself, and will require a wider evaluation on all the possible socio-economic and political factors, even if the data analysis of the Veneto situation could be used to revisit regional and central policies early on. If so, the regions are going to emulate the virtuous approach of Veneto, including more demanding requests to improve their diagnostic capacity that will weight on the central government.

## Figures and Tables

**Table 1:** Population (approximated) for Italy and Italian regions and for other countries included in the following analysis.

Countries/Regions	Overall Population	Database (Year)
Italy	60,359,546	Istituto Nazionale di Statistica—ISTAT (2019)
Lombardy	10,060,574	Istituto Nazionale di Statistica—ISTAT (2019)
Veneto	4,905,854	Istituto Nazionale di Statistica—ISTAT (2019)
Piedmont	4,356,406	Istituto Nazionale di Statistica—ISTAT (2019)
Spain	47,100,396	Istituto Nacional de Estadìstìca—INE (2019)
South Korea	51,629,512	Korean Statistical Information Service—KOSIS (November 2018)

**Table 2:** Values of *α*, *β*, *γ*, *δ*, *λ*, and *κ* describing SARS-CoV-2 outbreak in the recent literature.

Authors	Country/Region	Date	*α*	*β*	*γ*	*δ*	*λ*	*κ*
Peng et al. (2020) [3]	China without Hubei province	20 Jan–9 Feb	0.172	1	0.5	0.15	0.005–0.04	0.005–0.015
Peng et al. (2020)	Hubei province without Wuhan city	20 Jan–9 Feb	0.133	1	0.5	0.139	0.005–0.015	0.005–0.02
Peng et al. (2020)	Wuhan	20 Jan–9 Feb	0.085	1	0.5	0.135	0.005–0.015	0.005–0.03
Peng et al. (2020)	Beijing	20 Jan–9 Feb	0.175	0.99	0.5	0.175	0.005–0.04	0.002
Peng et al. (2020)	Shanghai	20 Jan–9 Feb	0.183	1	0.5	0.179	0.005–0.04	0
Calafiore et al. (2020) [5]	Italy	23 Feb–30 Mar		0.22			0.017	0.012
WHO report [10]	China	12 Feb		0.1–0.2				
Dandekar et al. (2020) [11]	Wuhan	1 Mar–1 Apr		1			0.023	
Dandekar et al. (2020)	Italy	1 Mar–1 Apr		0.74			0.032	
Dandekar et al. (2020)	South Korea	1 Mar–1 Apr		0.68			0.004	
Dandekar et al. (2020)	US	1 Mar–1 Apr		0.69			0.008	
Shaikh et al. (2020) [12]	India	14–26 Mar		0.59			0.1	
Lin et al. (2020) [13]	Wuhan	15 Jan–24 Feb		0.59–1.68	0.33		0.2	
Iwata et al. (2020) [14]	General case			0.1–1	0.07–0.5		0.1–1	

**Table 3:** Coefficients of the PSO best-solutions and deterministic solutions; bold refers to the best solution of the PSO approach (mean and variance on brackets), italic-bold refers to the solution given by the deterministic approach.

Country	*α*	*β* *	*γ*	*δ*	*λ*_0_	*λ*_1_	*κ*_0_	*κ*_1_	NRMSE
Italy	**0.021** (0.086, 0.004) ***0.012***	**0.510** (1.058, 0.200) ***1.170***	**0.265** (0.859, 0.226) ***1.065***	**0.103** (0.095, 0.01) ***0.020***	**0.017** (0.017, 6.3 × 10^−9^) ***0.017***	**2** (1.696, 0.180) ***1.983***	**0.029** (0.030, 2.7 × 10^−6^) ***0.033***	**0.038** (0.040, 3.8 × 10^−6^) ***0.043***	**0.035** ***0.043***
Spain	**0.037** (0.087, 0.002) ***0.026***	**1.777** (1.376, 0.082) ***2***	**0.946** (0.954, 0.198) ***0.154***	**0.238** (0.095, 0.004) ***0.614***	**0.044** (0.044, 6.8 × 10^−7^) ***0.043***	**0.156** (0.159, 0.0004) ***0.160***	**0.030** (0.030, 1.6 × 10^−6^) ***0.028***	**0.046** (0.047, 3.7 × 10^−6^) ***0.044***	**0.046** ***0.052***
South Korea	**0.292** (0.270 0.0004) ***0.1***	**2** (1.915, 0.009) ***0.974***	**2** (1.846, 0.046) ***1.902***	**0.123** (0.136, 7.8 × 10^−5^) ***0.313***	0.05 **	**-**	**8.3 × 10^−4^** (8.3 × 10^−4^, 3.5 × 10^−11^) ***0.007***	**7 × 10^−6^** (2.1 × 10^−6^, 2.1 × 10^−11^) ***0.134***	**0.074** ***0.078***
Lombardy	**0** (0.132, 0.012) ***8.9 × 10^−4^***	**0.460** (1.658, 0.188) ***0.81***	**0.295** (1.093, 0.531) ***0.302***	**0.145** (0.198, 0.065) ***0.253***	**0.027** (0.026, 3 × 10^−8^) ***0.027***	**0.981** (1.576, 0.247) ***1.925***	**0.036** (0.036, 6.9 × 10^−6^) ***0.045***	**0.031** (0.031, 6.9 × 10^−6^) ***0.0405***	**0.062** ***0.061***
Veneto	**0.133** (0.102, 0.002) ***0.049***	**1.704** (1.175, 0.190) ***0.97***	**0.920** (0.698, 0.144) ***0.246***	**0.032** (0.034, 0.0004) ***0.09***	**0.049** (0.182, 0.093) ***0.099***	**0.009** (0.008, 0.000) ***0.004***	**0.008** (0.008, 6.4 × 10^−8^) ***0.009***	**0.0215** (0.021, 1.3 × 10^−6^) ***0.024***	**0.035** ***0.040***
Piedmont	**0.240** (0.163, 0.009) ***0***	**1.990** (1.518, 0.232) ***0.994***	**0.265** (1.191, 0.374) ***0.195***	**0.012** (0.117, 0.052) ***0.344***	**0.386** (0.309, 0.104) ***0.069***	**0.001** (0.005, 2.5 × 10^−5^) ***0.007***	**0.019** (0.018, 3.7 × 10^−6^) ***0.019***	**0.034** (0.031, 1.9 × 10^−5^) ***0.035***	**0.056** ***0.050***

* The *β* parameter is time-dependent, as explained in the Methods section. The reported value is the initial value. It decreases by about 70% after mid-March, which is after 11 March national lockdown, with slight differences between Italian regions. This decrease is much less accentuated in the South Korea equation (approximately 10%); ** For South Korea, *λ*’s six model coefficients are not shown since they represent a different mathematical law from other countries, as explained in the Methods section. However, based on our fitting to the real data, it can be observed that *λ* gradually increases up to 0.5 at the mid of March; Numbers in bold are to distinguish the best solution of PSO method; Numbers in italics are to distinguish the deterministic approach solution.

## References

[1] Parham P.E., Michael E. (2011). Outbreak properties of epidemic models: The roles of temporal forcing and stochasticity on pathogen invasion dynamics. J. Theor. Biol..

[2] Li Q., Guan X., Wu P., Wang X., Zhou L., Tong Y., Ren R., Leung K.S.M., Lau E.H.Y., Wong J.Y. (2020). Early Transmission Dynamics in Wuhan, China, of Novel Coronavirus–Infected Pneumonia. N. Engl. J. Med..

[3] Peng L., Yang W., Zhang D., Zhuge C., Hong L. (2020). Epidemic analysis of COVID-19 in China by dynamical modeling. MedRxiv Epidemiol..

[4] Bacaër N. (2011). A Short History of Mathematical Population Dynamics.

[5] Calafiore G.C., Novara C., Possieri C. (2020). A Modified SIR Model for the COVID-19 Contagion in Italy. arXiv.

[6] COVID-19 Community Mobility Report. https://www.google.com/covid19/mobility.

[7] Kennedy J., Eberhart R. (1995). Particle swarm optimization. Proceedings of ICNN’95—International Conference on Neural Networks, Perth, WA, Australia, 27 November–1 December 1995.

[8] Shi P., Cao S., Feng P. (2020). SEIR Transmission dynamics model of 2019 nCoV coronavirus with considering the weak infectious ability and changes in latency duration. MedRxiv Infect. Dis. (Except HIV/AIDS).

[9] Cheynet E. Generalized SEIR Epidemic Model (Fitting and Computation). https://it.mathworks.com/matlabcentral/fileexchange/74545-generalized-seir-epidemic-model-fitting-and-computation.

[10] WHO 2019 Novel Coronavirus: Overview of the State of the Art and Outline of Key Knowledge Gaps/Slides. https://www.who.int/who-documents-detail/2019-novel-coronavirus-overview-of-the-state-of-the-art-and-outline-of-key-knowledge-gaps-slides.

[11] Dandekar R., Barbastathis G. (2020). Neural Network aided quarantine control model estimation of global Covid-19 spread. arXiv.

[12] Shaikh A.S., Shaikh I.N., Nisar K.S. (2020). A Mathematical Model of COVID-19 Using Fractional Derivative: Outbreak in India with Dynamics of Transmission and Control. Preprints.

[13] Lin Q., Zhao S., Gao D., Lou Y., Yang S., Musa S.S., Wang M.H., Cai Y., Wang W., Yang L. (2020). A conceptual model for the coronavirus disease 2019 (COVID-19) outbreak in Wuhan, China with individual reaction and governmental action. Int. J. Infect. Dis..

[14] Iwata K., Miyakoshi C. (2020). A Simulation on Potential Secondary Spread of Novel Coronavirus in an Exported Country Using a Stochastic Epidemic SEIR Model. JCM.

[15] Engelbrecht A.P. (2007). Computational Intelligence.

[16] Ratnaweera A., Halgamuge S.K., Watson H.C. (2004). Self-Organizing Hierarchical Particle Swarm Optimizer with Time-Varying Acceleration Coefficients. IEEE Trans. Evol. Comput..

[17] Pace F., Santilano A., Godio A. (2019). Particle swarm optimization of 2D magnetotelluric data. Geophysics.

[18] Pisano G.P., Sadun R., Zanini M. Lessons from Italy’s Response to Coronavirus. https://hbr.org/2020/03/lessons-from-italys-response-to-coronavirus.

[19] Wu X., Nethery R.C., Sabath B.M., Braun D., Dominici F. (2020). Exposure to air pollution and COVID-19 mortality in the United States: A nationwide cross-sectional study. MedRxiv Epidemiol..

